# Supplementary material for: A Critical View of the Application of the APEX Software (Aqueous Photochemistry of Environmentally-Occurring Xenobiotics) to Predict Photoreaction Kinetics in Surface Freshwaters
Source: Molecules. 2019 Dec 18;25(1):9. doi: 10.3390/molecules25010009 (PMC7017383; doi:10.3390/molecules25010009)
Supplement: Supplementary file 1 [file molecules-25-00009-s001.zip › 3.2.4_gcc-4.4.0.zip!/3.2.4_gcc-4.4.0/3.2.4_gcc-4.4.0/doc/octave/html/interpreter/Acknowledgements.html]

Acknowledgements - Untitled


Next: How You Can Contribute to Octave,
Up: Preface


---

### Acknowledgements

Many people have already contributed to Octave's development. The
following people have helped code parts of Octave or aided in
various other ways (listed alphabetically).

|  |  |  |
| --- | --- | --- |
| Ben Abbott | Andy Adler | Joel Andersson |
| Muthiah Annamalai | Shai Ayal | Roger Banks |
| Ben Barrowes | Alexander Barth | David Bateman |
| Heinz Bauschke | Karl Berry | David Billinghurst |
| Don Bindner | Jakub Bogusz | Moritz Borgmann |
| Richard Bovey | Marcus Brinkmann | Remy Bruno |
| Marco Caliari | Daniel Calvelo | John C. Campbell |
| Jean-Francois Cardoso | Joao Cardoso | Larrie Carr |
| David Castelow | Vincent Cautaerts | Clinton Chee |
| Albert Chin-A-Young | Carsten Clark | J. D. Cole |
| Martin Costabel | Michael Creel | Jeff Cunningham |
| Martin Dalecki | Jorge Barros de Abreu | Carlo de Falco |
| Thomas D. Dean | Philippe Defert | Bill Denney |
| David M. Doolin | Pascal A. Dupuis | John W. Eaton |
| Dirk Eddelbuettel | Paul Eggert | Stephen Eglen |
| Peter Ekberg | Rolf Fabian | Stephen Fegan |
| Ramon Garcia Fernandez | Torsten Finke | Jose Daniel Munoz Frias |
| Castor Fu | Eduardo Gallestey | Walter Gautschi |
| Klaus Gebhardt | Driss Ghaddab | Nicolo Giorgetti |
| Michael Goffioul | Glenn Golden | Tomislav Goles |
| Keith Goodman | Brian Gough | Steffen Groot |
| Etienne Grossmann | Peter Gustafson | Kai Habel |
| William P. Y. Hadisoeseno | Jaroslav Hajek | Benjamin Hall |
| Kim Hansen | Soren Hauberg | Dave Hawthorne |
| Daniel Heiserer | Martin Helm | Stefan Hepp |
| Yozo Hida | Ryan Hinton | Roman Hodek |
| A. Scottedward Hodel | Richard Allan Holcombe | Tom Holroyd |
| David Hoover | Kurt Hornik | Christopher Hulbert |
| Cyril Humbert | Teemu Ikonen | Alan W. Irwin |
| Geoff Jacobsen | Mats Jansson | Cai Jianming |
| Steven G. Johnson | Heikki Junes | Atsushi Kajita |
| Jarkko Kaleva | Mohamed Kamoun | Lute Kamstra |
| Thomas Kasper | Joel Keay | Mumit Khan |
| Paul Kienzle | Aaron A. King | Arno J. Klaassen |
| Geoffrey Knauth | Heine Kolltveit | Ken Kouno |
| Oyvind Kristiansen | Piotr Krzyzanowski | Volker Kuhlmann |
| Tetsuro Kurita | Miroslaw Kwasniak | Rafael Laboissiere |
| Kai Labusch | Claude Lacoursiere | Walter Landry |
| Bill Lash | Dirk Laurie | Maurice LeBrun |
| Friedrich Leisch | Timo Lindfors | Benjamin Lindner |
| Ross Lippert | David Livings | Erik de Castro Lopo |
| Massimo Lorenzin | Emil Lucretiu | Hoxide Ma |
| James Macnicol | Jens-Uwe Mager | Ricardo Marranita |
| Orestes Mas | Makoto Matsumoto | Tatsuro Matsuoka |
| Laurent Mazet | G. D. McBain | Alexander Mamonov |
| Christoph Mayer | Thorsten Meyer | Petr Mikulik |
| Stefan Monnier | Antoine Moreau | Kai P. Mueller |
| Victor Munoz | Carmen Navarrete | Todd Neal |
| Al Niessner | Rick Niles | Takuji Nishimura |
| Kai Noda | Eric Norum | Krzesimir Nowak |
| Michael O'Brien | Peter O'Gorman | Thorsten Ohl |
| Arno Onken | Luis F. Ortiz | Scott Pakin |
| Gabriele Pannocchia | Sylvain Pelissier | Per Persson |
| Primozz Peterlin | Jim Peterson | Danilo Piazzalunga |
| Nicholas Piper | Robert Platt | Hans Ekkehard Plesser |
| Tom Poage | Orion Poplawski | Ondrej Popp |
| Jef Poskanzer | Francesco Potorti | James B. Rawlings |
| Eric S. Raymond | Balint Reczey | Michael Reifenberger |
| Jason Riedy | Petter Risholm | Matthew W. Roberts |
| Andrew Ross | Mark van Rossum | Kevin Ruland |
| Ryan Rusaw | Olli Saarela | Toni Saarela |
| Juhani Saastamoinen | Radek Salac | Ben Sapp |
| Aleksej Saushev | Alois Schloegl | Michel D. Schmid |
| Julian Schnidder | Nicol N. Schraudolph | Sebastian Schubert |
| Ludwig Schwardt | Thomas L. Scofield | Daniel J. Sebald |
| Dmitri A. Sergatskov | Baylis Shanks | Joseph P. Skudlarek |
| John Smith | Julius Smith | Shan G. Smith |
| Joerg Specht | Quentin H. Spencer | Christoph Spiel |
| Richard Stallman | Russell Standish | Doug Stewart |
| Jonathan Stickel | Thomas Stuart | Ivan Sutoris |
| John Swensen | Ariel Tankus | Georg Thimm |
| Duncan Temple Lang | Kris Thielemans | Olaf Till |
| Thomas Treichl | Frederick Umminger | Utkarsh Upadhyay |
| Stefan van der Walt | Peter Van Wieren | James R. Van Zandt |
| Gregory Vanuxem | Ivana Varekova | Thomas Walter |
| Olaf Weber | Thomas Weber | Rik Wehbring |
| Bob Weigel | Andreas Weingessel | Michael Weitzel |
| Fook Fah Yap | Michael Zeising | Federico Zenith |
| Alex Zvoleff |  |  |

Special thanks to the following people and organizations for
supporting the development of Octave:

- The United States Department of Energy, through grant number
  DE-FG02-04ER25635.- Ashok Krishnamurthy, David Hudak, Juan Carlos Chaves, and Stanley
    C. Ahalt of the Ohio Supercomputer Center.- The National Science Foundation, through grant numbers CTS-0105360,
      CTS-9708497, CTS-9311420, CTS-8957123, and CNS-0540147.- The industrial members of the Texas-Wisconsin Modeling and Control
        Consortium (TWMCC).- The Paul A. Elfers Endowed Chair in Chemical Engineering at the
          University of Wisconsin-Madison.- Digital Equipment Corporation, for an equipment grant as part of their
            External Research Program.- Sun Microsystems, Inc., for an Academic Equipment grant.- International Business Machines, Inc., for providing equipment as part
                of a grant to the University of Texas College of Engineering.- Texaco Chemical Company, for providing funding to continue the
                  development of this software.- The University of Texas College of Engineering, for providing a
                    Challenge for Excellence Research Supplement, and for providing an
                    Academic Development Funds grant.- The State of Texas, for providing funding through the Texas
                      Advanced Technology Program under Grant No. 003658-078.- Noel Bell, Senior Engineer, Texaco Chemical Company, Austin Texas.- John A. Turner, Group Leader, Continuum Dynamics (CCS-2), Los Alamos
                          National Laboratory, for registering the octave.org domain name.- James B. Rawlings, Professor, University of Wisconsin-Madison,
                            Department of Chemical and Biological Engineering.- Richard Stallman, for writing GNU.

This project would not have been possible without the GNU software used
in and to produce Octave.
